# Evaluating the Impact of Different Treatments on the Quality of Life in Patients With Burning Mouth Syndrome: A Scoping Review

**DOI:** 10.7759/cureus.70419

**Published:** 2024-09-29

**Authors:** João Mendes Abreu, Anabela Quitério, Érica Cerqueira, Rita Ribeiro, Tiago Nunes, José Pedro Figueiredo, Ana Corte Real

**Affiliations:** 1 Faculty of Medicine, University of Coimbra, Coimbra, PRT; 2 Stomatology Service - Head, Neck, and Skin Surgery Department, Unidade Local de Saúde (ULS) de Coimbra, Coimbra, PRT; 3 Coimbra Hospital and University Centre, Clinical and Academic Centre of Coimbra, Coimbra, PRT; 4 Maxillofacial Service - Head, Neck, and Skin Surgery Department, Unidade Local de Saúde (ULS) de Coimbra, Coimbra, PRT

**Keywords:** burning mouth syndrome, clonazepam, health-related quality of life, low-level laser therapy, oral health, quality of life (qol)

## Abstract

The profound impact of burning mouth syndrome (BMS) on patients' quality of life (QoL) highlights the critical need to identify effective treatments for this condition. This study aims to evaluate and compare the health-related quality of life (HRQoL) and oral health-related quality of life (OHRQoL) among individuals diagnosed with BMS, focusing on different treatment modalities.

For that purpose, a scoping review was designed following the Preferred Reporting Items for Systematic Reviews and Meta-Analyses (PRISMA) for scoping review reporting guidelines and the registration with the International Prospective Register of Systematic Reviews (PROSPERO). An electronic search was then conducted in March 2024, encompassing the following databases: PubMed, Embase, Cochrane, Web of Science, and Trip Database. Publications were deemed eligible if they assessed the impact of different treatments for BMS on health-related and oral health-related QoL.

Out of the initial 5400, only 13 studies were considered suitable to be included in this review. The instrument used to evaluate HRQoL was the 36-Item Short Form Survey (SF-36). For OHRQoL, the preferred tools were the Oral Health Impact Profile (OHIP) and the Geriatric Oral Health Assessment Index (GOHAI). Literature reported improvements in patients' HRQoL across the majority of analyzed treatment modalities. However, low-level laser therapy (LLLT) and n-acetylcysteine (NAC) plus clonazepam were the most effective in improving OHRQoL.

This review highlights several promising treatment options for improving both HRQoL and OHRQoL in individuals with BMS. Nevertheless, the variability among the studies analyzed underscores the need for further research to identify and establish consistently effective treatments for this condition, reflecting the need for consistent trial designs to accurately assess the true impact of treatments on the disease.

## Introduction and background

Burning mouth syndrome (BMS) is a chronic condition defined as an intraoral burning feeling, pain, or dysesthesia without visible oral mucosal changes or abnormal radiologic or laboratory findings. The diagnosis also implies the persistence of symptoms for at least two hours daily over a minimum three-month period [1,2]. With a global prevalence of 1.73%, which can be up to three times higher in certain populations such as Europeans, BMS primarily affects peri- and postmenopausal females [3,4].

The hallmark of BMS is therefore a burning sensation or pain, which may be localized to the tongue and/or lips or more widespread, affecting the entire oral cavity. It is also described that the severity of pain tends to increase toward the end of the day or in response to specific foods [2]. Burning mouth may also be a symptom of other diseases, especially when local or systemic factors are involved, such as vitamin and mineral deficiencies, menopause, endocrine disorders (e.g., diabetes mellitus), drug-induced xerostomia, and allergic-like reactions (e.g., dental prosthesis). However, in cases where no underlying dental or medical etiologies are identified and oral manifestations are absent, the term BMS should be used [3,5].

The pathogenesis of BMS remains under discussion within the scientific community. Understanding the relative involvement of peripheral versus central mechanisms in BMS pain has important implications concerning the setting of diverse treatment approaches [2,6,7]. Therefore, some authors have chosen to classify BMS into two subtypes: central and peripheral, with the central subtype often associated with psychiatric comorbidities (e.g., depression or anxiety) and unresponsiveness to local treatments, in contrast to the peripheral subtype, which responds well to peripheral lidocaine blocks and topical clonazepam and is not associated with any systemic conditions [8]. Consequently, given the incomplete understanding of BMS etiology and mechanisms, developing an effective treatment protocol has posed a considerable challenge [6,9,10].

Health-related quality of life (HRQoL) and oral health-related quality of life (OHRQoL) are widely accepted concepts focusing on personal multilevel domains and their impact on individuals concerning general and oral health issues [11,12]. The 36-Item Short Form Survey (SF-36) is one of the most well-recognized HRQoL assessment tools. It includes 36 items selected from a larger pool used in the RAND Corporation's Medical Outcomes Study (MOS) [13,14]. Likewise, the Oral Health Impact Profile (OHIP) is a similar instrument, although specifically developed to measure people's perception of the social impact of oral disorders on their well-being. Due to the extensive nature of the original questionnaire with 49 items (OHIP-49), an abbreviated version known as the OHIP-14 was developed [15,16]. Another noteworthy OHRQoL instrument is the Geriatric Oral Health Assessment Index (GOHAI), designed for assessing oral health among elderly individuals and its influence on their holistic well-being [17].

Research suggests that individuals with BMS typically experience poorer overall health, increased comorbidities, higher medication use, and worse outcomes in health-related measures [18]. Despite the significant impact of BMS, confidence in the effectiveness of available treatments remains low due to the lack of efficacy of available treatments [6,9,10]. Therefore, this review aims to assess and compare the impact of different treatments on HRQoL and/or OHRQoL in patients with BMS. To this end, the authors have chosen to conduct a scoping review in an attempt to include a larger dataset encompassing all types of treatments and HRQoL and/or OHRQoL measurement tools.

## Review

Materials and methods

Study Design and Search Strategy

This scoping review was registered in the International Prospective Register of Systematic Reviews (PROSPERO) (CRD42022309010) and was prepared following the Preferred Reporting Items for Systematic Reviews and Meta-Analyses (PRISMA) for scoping review checklist [19]. The primary objective was to identify studies examining the impact of various treatments on HRQoL and OHRQoL in patients with BMS. This was conducted using a structured Population, Intervention, Comparison, and Outcome (PICO) framework: P, adult patients diagnosed with BMS; I, various treatments for BMS; C, patients receiving no treatment, a placebo, or a predetermined treatment; O, impact on HRQoL and/or OHRQoL (Table 1). A comprehensive search was conducted across major scientific databases (PubMed, Cochrane, Embase, Web of Science, and Trip Database) using the predetermined search terms and strategies, covering publications up to March 1, 2024 (Table 2).

**Table 1:** PICO framework BMS, burning mouth syndrome; HRQoL, health-related quality of life; OHRQoL, oral health-related quality of life

PICO	Inclusion criteria
Population	Adult patients diagnosed with BMS
Intervention	Various treatments for BMS
Comparison	Patients receiving no treatment, a placebo, or a predetermined treatment
Outcome	Impact on HRQoL and OHRQoL

**Table 2:** Search terms and strategy BMS, burning mouth syndrome; MeSH, Medical Subject Heading

Topic	Search terms and strategy
BMS	("Burning Mouth Syndrome" [MeSH] OR "Glossalgia" [MeSH]) OR stomatodynia OR glossodynia OR oral dysaesthesia OR orodynia OR glossopyrosis OR burning tongue OR burning tongue syndrome OR stomatopyrosis OR sore tongue OR sore mouth OR painful tongue OR burning lips syndrome OR scalded mouth syndrome
AND	
Quality of life	"Quality of Life" [MeSH] OR well being OR well-being OR general well being OR general well-being OR oral health related quality of life OR oral health-related quality of life OR health related quality of life OR health-related quality of life OR health OR welfare OR wellness
AND	
Treatment	"Therapeutics" [MeSH] OR therapy OR medication OR medicaments OR cure OR medicine OR care OR remedy OR healing

Eligibility Criteria, Study Selection, and Data Extraction

Studies were considered eligible if they met the following inclusion criteria: (i) observational studies (cross-sectional and case-control) or interventional studies (randomized controlled trials (RCT) and non-randomized control trials (N-RCT)); (ii) adult patients aged 18 and above diagnosed with BMS; (iii) written in Portuguese, English, Spanish, or French; (iv) testing any treatment for BMS; and (v) assessed HRQoL or OHRQoL. Studies were excluded if they met the following exclusion criteria: (i) conducted in patients without diagnostic criteria of BMS, (ii) did not assess HRQoL or OHRQoL, (iii) did not provide individualized data for BMS, (iv) lacked full-text availability, and (v) published in a format other than that specified in (i) of the inclusion criteria and did not consist of original research.

The removal of duplicates and the selection of articles relevant to this analysis were conducted using the reference management software "Rayyan" [20]. Two independent reviewers assessed the remaining abstracts, and any uncertainties regarding study inclusion or exclusion were resolved by a third reviewer. Subsequently, both researchers reviewed the remaining articles to determine their eligibility based on the criteria defined by the PICO framework. For all studies included, the following data were extracted and summarized: (i) authors and year of publication, (ii) study design, (iii) sample size, (iv) epidemiological information, (v) treatment used, (vi) applied questionnaire, (vii) findings, and (viii) conclusions.

Quality Assessment

The scientific quality of the studies selected was assessed independently by two reviewers using two tools: RoB 2 [21] for RCT and ROBINS-I [22] for N-RCT. Subsequently, for each study, the overall risk of bias was determined according to the preestablished criteria and categorized from low to high. Following this, the robvis tool was used to summarize the results [23]. If assessment outcomes were conflicting, a consensus-based final score was attributed.

Results

Study Selection and Description and Risk of Bias

**Figure 1:**
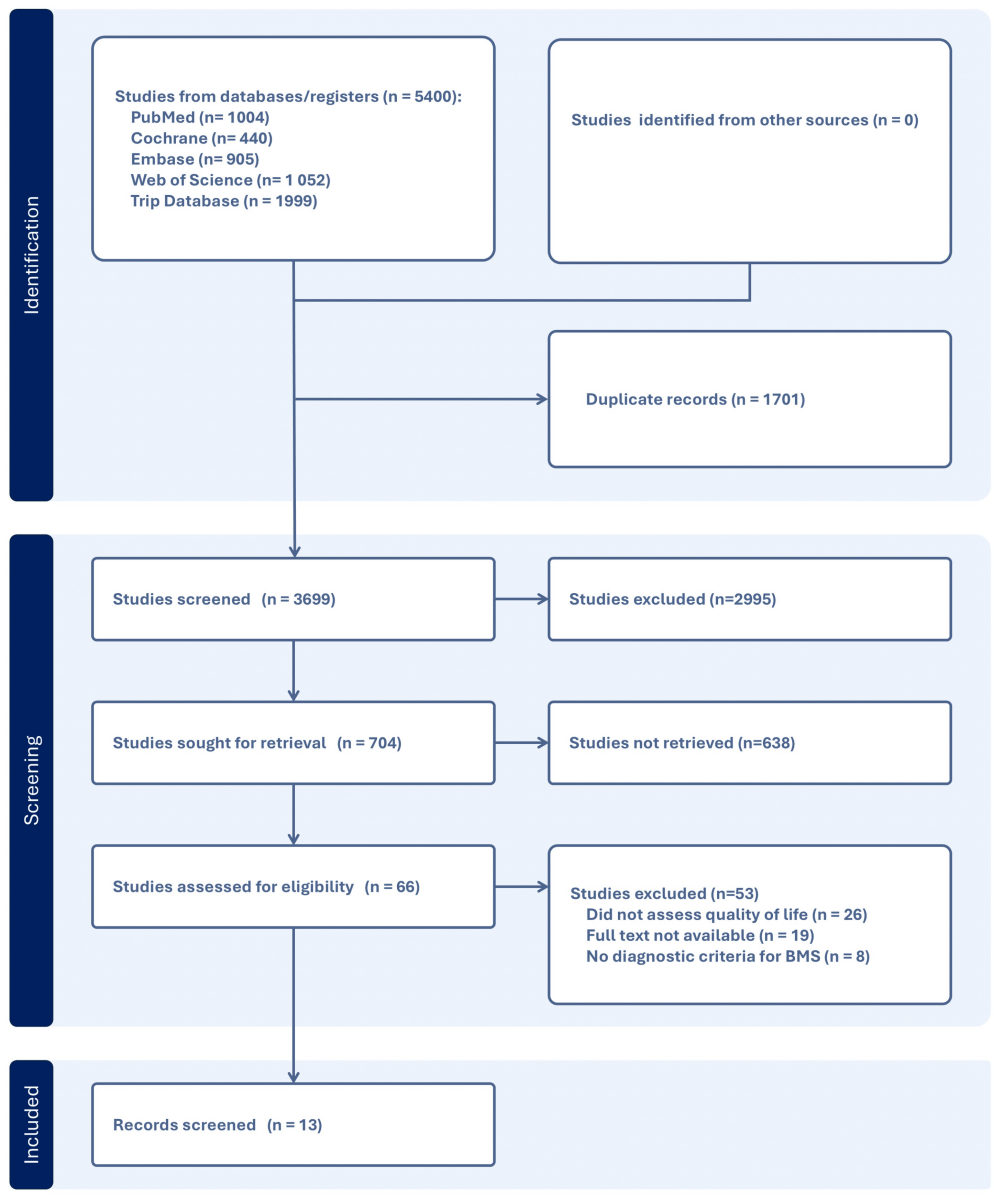
PRISMA flowchart describing article selection PRISMA, Preferred Reporting Items for Systematic Reviews and Meta-Analyses; BMS, burning mouth syndrome

Out of an initial selection of 5400 articles, 13 were chosen for descriptive analysis (Figure 1). Among these, nine articles were RCT [24-32], two were N-RCT [33,34], and two were case series [35,36]. The characteristics of the included studies are summarized and provided in the Appendices.

**Figure 2:**
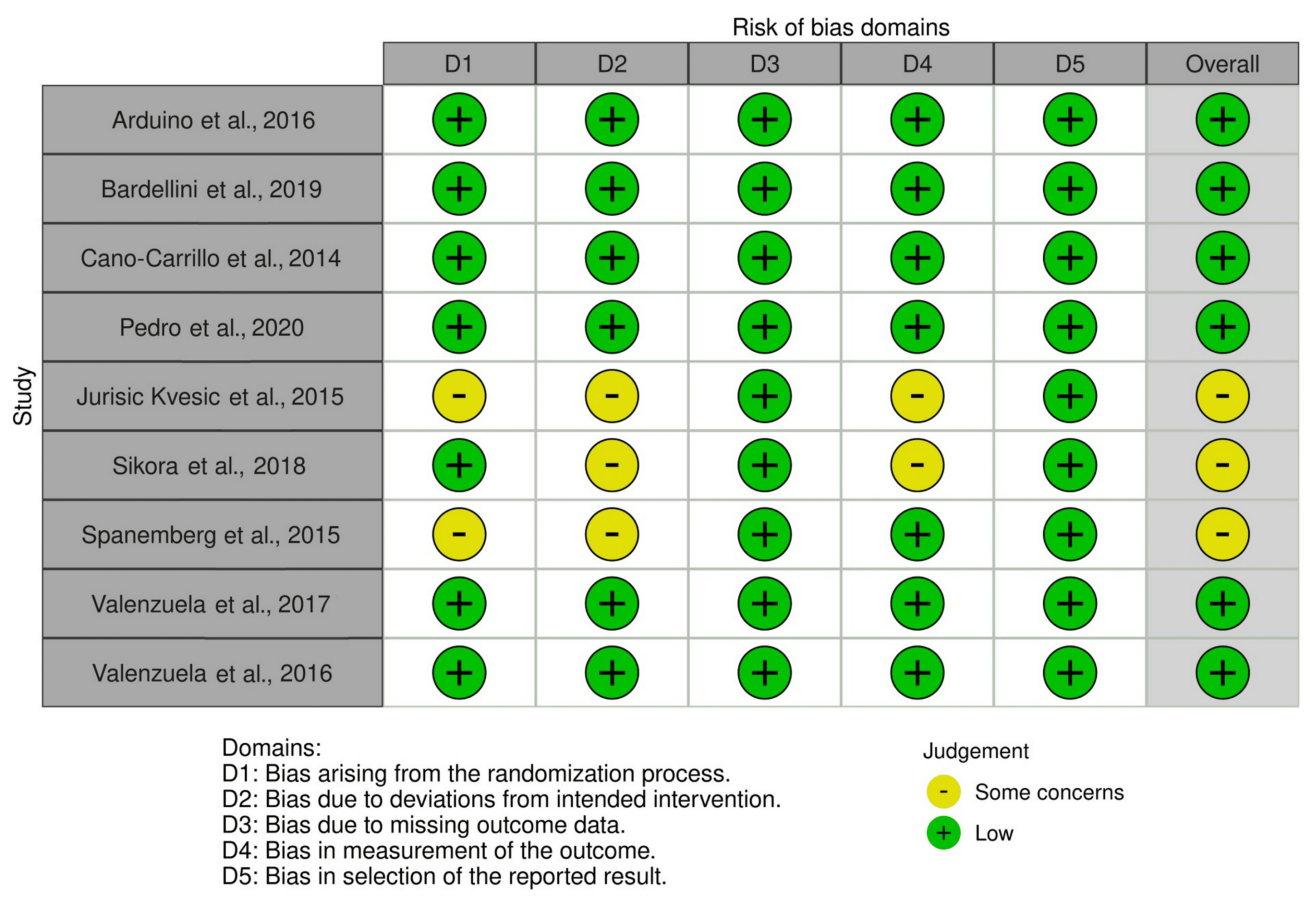
Quality assessment for randomized controlled trials References: Arduino et al. [24], Bardellini et al. [25], Cano-Carrillo et al. [26], de Pedro et al. [27], Jurisic Kvesic et al. [28], Sikora et al. [29], Spanemberg et al. [30], Valenzuela and Lopez-Jornet [31], and Valenzuela et al. [32]

**Figure 3:**
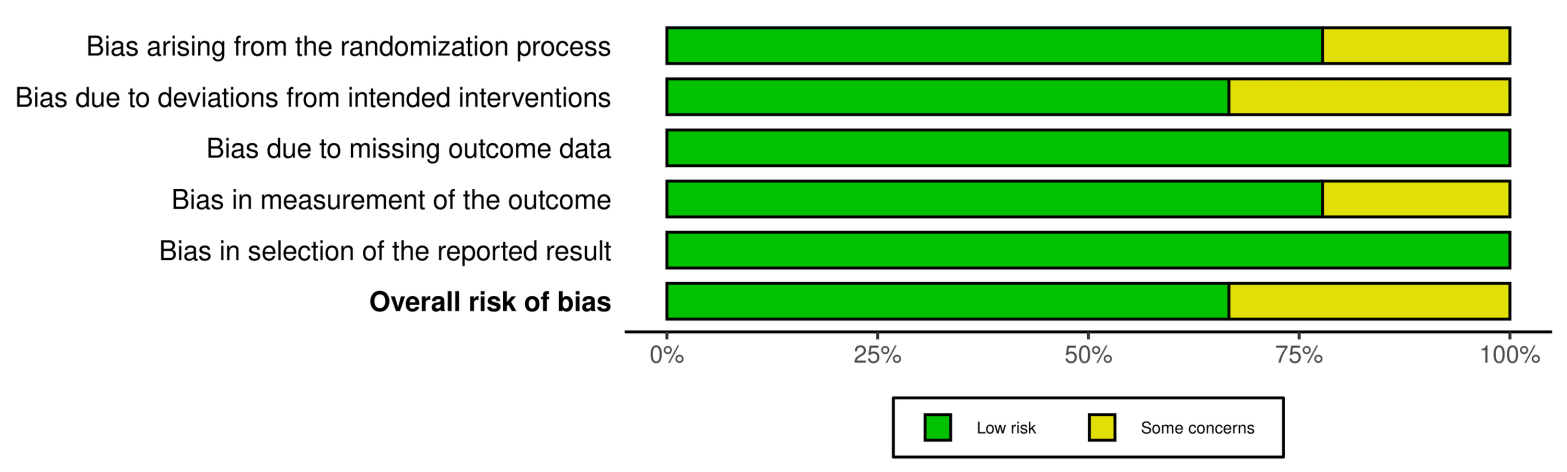
Visual representation of quality assessment for randomized controlled trials

The potential for bias in this study was evaluated using the RoB 2 tool for RCT [21] and the ROBINS-I tool, which was adapted for N-RCT [22], with the aid of the robvis tool [23] for visual representation. Out of the nine RCTs, six were found to present a low risk of bias across all domains. However, three studies raised some concerns, particularly in relation to the randomization process, deviations from the intended intervention, and the measurement of the outcome (Figures 2, 3). These results may potentially impact the reliability of these specific trials.

**Figure 4:**
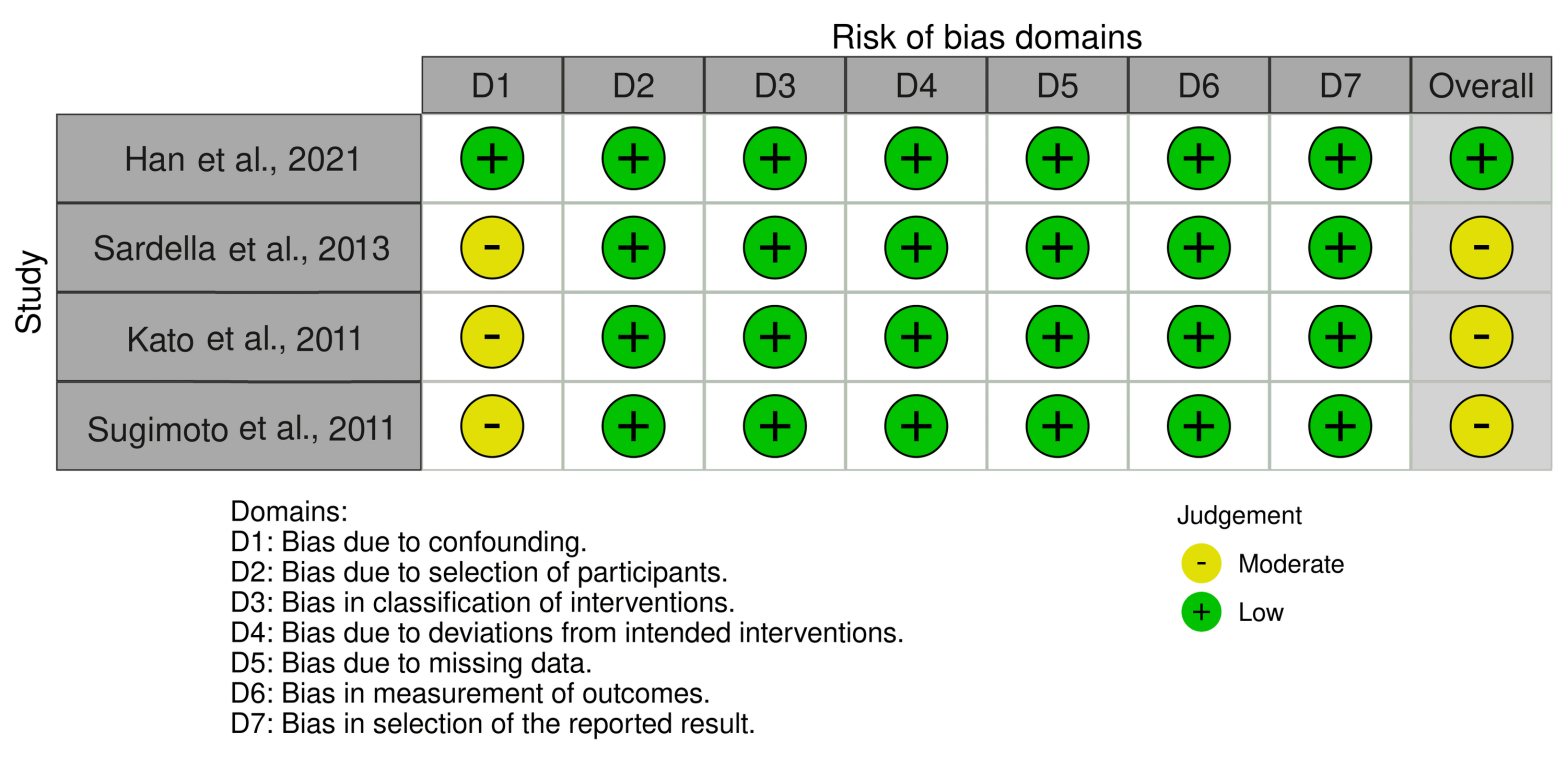
Quality assessment for non-randomized controlled trials References: Han et al. [33], Sardella et al. [34], Kato et al. [35], and Sugimoto [36]

**Figure 5:**
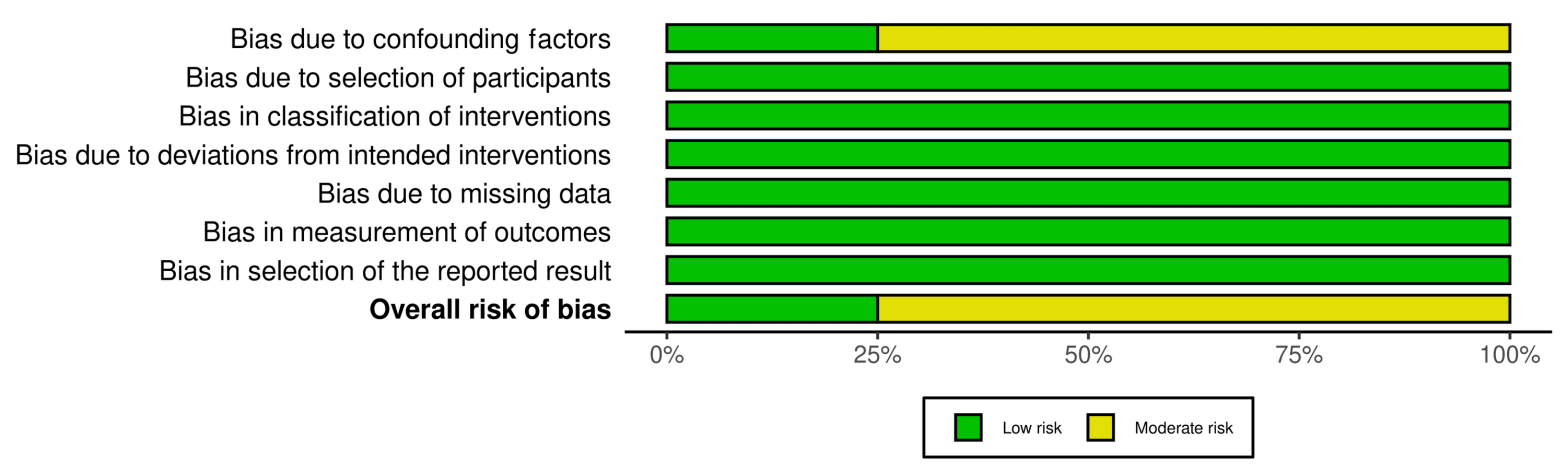
Visual representation of quality assessment for non-randomized controlled trials

Among the four N-RCTs reviewed, one exhibited a low risk of bias. In contrast, three studies were found to have a moderate risk, primarily due to the presence of confounding factors (Figures 4, 5). These confounders may have affected the study outcomes, thus posing a moderate threat to the internal validity of these trials, though not rising to the level of high risk.

Characteristics of the Participants

A total of 701 individuals were evaluated across the included studies. The diagnostic criteria predominantly considered for BMS were "oral burning sensation continuous throughout the day lasting for more than six months without the detection of oral mucosal lesions" [24-26,28-30,34] and the diagnostic criteria from the International Classification of Headache Disorders, third edition (ICHD-3) (International Headache Society, 2018) [27,31-33]. One trial used the International Association for the Study of Pain definition [35], while another [36] selected patients based on criteria supplied by the literature. The participants were recruited from dental clinics at the same institutes, with 90.15% (n=632) of the patients being female with ages ranging from 20 to 88 years old. The patients originated from the following countries: Spain [26,27,31,32], Italy [24,25,34], Croatia [28,29], Japan [35,36], Brazil [30], and Korea [33].

Outcome Measures

The quality of life (QoL) was assessed inconsistently across the publications. The instrument used for the evaluation of HRQoL was the SF-36. The SF-36 assesses eight different health concepts using multi-item scales (35 items), as well as the patient's perceived health during the last 12 months. Scores for each of these dimensions range from 0 to 100. Higher scores indicate higher HRQoL [13,14]. For OHRQoL, the preferred tools were the OHIP-14 or OHIP-49 [15,16] and the GOHAI [17]. The OHIP assesses the social impacts of oral disorders. The questionnaire evaluates dysfunction, discomfort, and disability caused by oral disorders, according to seven dimensions. For each question, the patients were asked how frequently they had experienced the impact in the preceding 12 months. Responses are coded on a five-point Likert scale (0-5) with higher OHIP scores indicating worse OHRQoL [15,16]. The GOHAI has been used to evaluate the physical, physiological, and psychological aspects of oral health, thus aiding routine clinical assessment. It consists of 12 closed-ended questions evaluating self-perceived oral health. The response to each question was assessed using a four-point Likert scale (1-4). A high score means better OHRQoL [17]. Due to the heterogeneity among the selected studies, the statistical analysis of the results was not conducted.

Characteristics of the Interventions and Their Effect on the Quality of Life

Low-level laser therapy (LLLT): Low-level laser therapy (LLLT) was the most frequently evaluated treatment, in a total of six trials. In general, its effects were compared to a placebo, except for one study, which compared it to clonazepam [24]. One trial studied the effect of LLLT once a week for 10 weeks versus a placebo [25]. The laser was used with discontinuous combined wavelengths between 660 and 970 nm, medium power of 3.2 W (6.4 W pulsed at 50%), treatment time of three minutes and 51 seconds, frequency of 1-20000 Hz, and spot size 1 cm^2^. In group A (LLLT), the OHIP-14 score decreased from 16.09±4.2 to 7.34±3.78 at the one-month follow-up. In group B (placebo), the OHIP-14 went from 15.26±3.75 to 10.43±2.99. Therefore, the patients treated with laser had a statistically significant improvement in OHRQoL, compared to the placebo group.

de Pedro et al. conducted a study with a diode laser with a wavelength of 810 nm (1.2 W/cm^2^, 10 seconds) in 56 points in the oral cavity with a 2 mm distance between them [27]. The sessions took place twice a week for five weeks consecutively. The improvement in OHRQoL was modest in the laser therapy group, as indicated by a slight reduction in OHIP-14 scores, from 16.5±13.09 to 12.5±7.10, and increasing in the placebo group, from 21.30±9.07 to 24.10±11.50. No significant differences were also observed in the HRQoL SF-36 questionnaire.

In another study, a GaAlAs laser (830 nm, 100 mW, 12 J/cm^2^) was used during 10 sessions over 10 days [29]. There were no significant differences between the treatment and the placebo groups regarding the OHRQoL assessed with the OHIP-14 score.

Spanemberg et al. compared three different laser protocols with a control group [30]. One group received GaAlAs infrared laser weekly (830 nm, 100 mW, 5 J, 176 J/cm^2^, 50 seconds) for 10 weeks. Another group received treatment three times a week for three weeks, with GaAlAs laser set to the same parameters. The third group was the red laser group, receiving treatment with an InGaAlP laser (685 nm, 35 mW, 2 J, 72 J/cm^2^, 58 seconds) three times a week for three weeks. All groups showed a statistically significant decrease in the OHIP-14 scores. However, only the infrared laser group differed significantly from the control group regarding OHRQoL (OHIP-14 went from 12.87±7.78 to 6.89±4.05 in the treatment group, while in the control group, it went from 12.87±7.78 to 6.89±4.05).

Valenzuela and Lopez-Jornet used a GaAlAs laser with 815 nm of wavelength in two groups and compared it to a placebo group: Group I received 1 W output power, continuous emissions, for four seconds, 4 J, and fluence rate of 133.3 J/cm^2^, and in group II, the same laser was used but for six seconds, 6 J, and fluence rate of 200 J/cm^2 ^[31]. All groups received a weekly dose for four weeks. LLLT significantly enhanced OHIP-14 values from the initial evaluation in groups I (29.88±3.6 to 28.50±3.1) and II (from 29.56±5.9 to 28.25±6.1) compared to the placebo group (from 29.33±5.9 to 29.25±6.3).

Arduino et al. [24] designed a study that aimed to compare LLLT to clonazepam. The laser group received two sessions weekly for five weeks, with an AlGaAs diode laser (980 nm, 300 mW, 10 J/cm^2^). Subjects submitted to LLLT reported a greater reduction in pain sensation in all studied variables. OHIP-49 score also went from 59.28±37.95 (baseline) to 48.22±32.11 after 12 weeks of treatment, a statistically significant result.

Lycopene-enriched virgin olive oil and topical chamomile: In one study, lycopene-enriched virgin olive oil (300 parts per million {ppm}) was topically applied at a dose of 1.5 mL, three times/day, for 12 weeks, and compared to a placebo [26]. No significant differences were found between the groups with the OHIP-14 score decreasing from 21 to 18 in the treatment group and from 23.5 to 18 in the placebo group. Consistently, the SF-36 questionnaire also failed to present significant differences regarding the evolution of HRQoL.

Valenzuela et al. compared chamomile gel (2%) applied topically twice a day, for one month, to a placebo [32]. Both treatments showed equivalent results, thus calling into question the validity of chamomile gel (2%) for the management of BMS.

Milnacipran: Milnacipran was tested in two trials. One trial started at a prescribed dose of 15 mg/day and escalated to 60 mg/day after 28 days, for a total of 84 days (12 weeks). GOHAI and SF-36 scores did not change before and after treatment [35]. In another trial, a 12-week dose-escalation study was conducted in 56 female patients. The initial dosage of milnacipran was 30 mg/day, and the dosage was raised to 60 mg and 90 mg/day at four and eight weeks. No significant differences were found in most of the SF-36 items except bodily pain [36].

Acupuncture: Two studies investigated the impact of acupuncture on HRQoL. In one trial, 20 participants were treated over four weeks, three times per week [28]. This group was compared to another group of 22 patients taking clonazepam (the results described in the next topic). There were statistically significant changes in all outcome measures, with the SF-36 score increasing from 71.6±4.5 to 85.6±5.2 in the acupuncture group. However, no significant differences were seen between the two therapeutic regimens. In another study, Sardella et al. performed an eight-week acupuncture treatment, consisting of 20 sessions [34]. No significant improvement in HRQoL was observed, measured by the SF-36 test.

Clonazepam: In the analyzed studies, the effect of clonazepam on HRQoL and OHRQoL was evaluated in comparison to LLLT, acupuncture, and N-acetylcysteine (NAC), all of which are already partially addressed [24,28,33]. In the Arduino et al. study, one group was treated with topical clonazepam, applied directly to the affected area without ingestion, for three minutes, three times a day for 21 days [24]. The results showed less favorable results (compared to the laser group) with an OHIP-49 (63.47±33.69 to 67.87±42.97). In the trial comparing acupuncture to clonazepam, a group of 22 patients took clonazepam 0.5 mg/day for two weeks, and, after two weeks, 1 mg/day was taken for the next two weeks [28]. There was a significant change in the score of SF-36 (from 73.4±5.2 to 86±4.7). In the study comparing the effect of NAC and clonazepam, the authors divided the subjects into three groups: one receiving NAC (400 mg/day), another receiving clonazepam (0.5 mg/day), and a third receiving both NAC and clonazepam [33]. OHIP-14 scores significantly decreased in all groups after the eight-week treatments with the NAC/clonazepam combination therapy proving to be superior to either monotherapy.

Discussion

Given the enduring impact of BMS on QoL, finding effective treatments is essential. The range of treatments assessed in the selected studies for this review indicates that most aim to address the suspected underlying causes of BMS. However, these interventions frequently lack support from controlled studies [6].

Numerous reviews have investigated the efficacy of several treatment modalities for BMS, evaluating their effect on factors such as symptom reduction [2,6,7,9,10]. However, despite long-standing concerns about the effect of BMS on patient QoL, there is a need to assess how a range of different treatments affect both HRQoL and OHRQoL, providing the rationale for this scoping review.

Several instruments have been validated to measure HRQoL, the SF-36 being one of the most frequently used [11]. The SF-36 measures two distinct concepts: a physical dimension, represented by the physical component summary (PCS), and a mental dimension, represented by the mental component summary (MCS). All scales contribute in different proportions to the scoring of both PCS and MCS measures [13,14,37].

The OHIP and the GOHAI are regarded as two of the most comprehensive assessments for measuring the OHRQoL [15-17,38]. OHIP is a questionnaire evaluating seven dimensions, whose original version was condensed to the 14 most significant items [15,16,39]. GOHAI includes 12 items that measure three dimensions, with higher scores indicating better oral health [17,40]. Nevertheless, these measurement instruments may lack objectivity and reflect factors such as the emotional component of the pathology [41].

Out of the 13 selected studies, six measured HRQoL, all of which used the SF-36 [26-28,34-36]. For nearly all treatment types under analysis, the results showed nonsignificant statistical differences. The study conducted by Jurisic Kvesic et al. was the only one reporting a significant change in SF-36 scores for both the clonazepam and acupuncture groups [28]. However, no significant differences were observed between the two therapeutic regimens. These findings align with existing literature, showing that no single treatment consistently yields superior results. Instead, improvements in symptoms may not always correspond with enhancements in HRQoL [26]. OHRQoL was also assessed in 10 of the 13 studies, translating an increasing interest in the measurement of the practical and psychosocial repercussions of oral illnesses on QoL [24-27,29-33,36]. The majority of the studies opted for the OHIP-14 or OHIP-49 instruments [24-27,29-33], with only one utilizing GOHAI [36].

In three out of five studies treating patients with LLLT, a significant improvement in OHRQoL was verified [25,30,31]. In another study, only a slight improvement was observed in the laser group [27], while only one trial reported no significant differences between groups [29]. Arduino et al. ascertained that clonazepam yielded less favorable outcomes compared to the laser group in terms of OHRQoL, as assessed using OHIP-49 [24]. The combination therapy of NAC plus clonazepam proved to be more effective. However, neither monotherapy had significant improvement in OHRQoL as measured through OHIP-14 [33]. Topical chamomile and milnacipran did not influence OHRQoL [32,35,36]. The studies testing acupuncture did not measure its impact on OHRQoL [28,34].

Limitations

Although all studies evaluated the QoL, it is crucial to note that no strict evaluation can be made among the treatments tested. This restriction arises due to the lack of uniform diagnostic criteria, which may lead to relevant variations in the study population and discrepancies in the identification of outcomes. Another limiting factor was the heterogeneous treatment protocols used, as well as the inclusion of RCT and N-RCT, which made it impossible to compare the results obtained concerning the multitude of confounding factors. Lastly, not every study evaluated both HRQoL and OHRQoL, and there was a lack of long-term assessments, which restricts the conclusions that can be drawn.

## Conclusions

While some treatments have shown apparent efficacy, the inconsistency of the evidence highlights the need for further research to identify a definitive treatment for BMS that not only reduces symptoms but also improves patients' QoL, HRQoL, and OHRQoL. Consistent trial designs are therefore essential to enable more accurate comparisons between treatments, particularly in terms of their impact on HRQoL and OHRQoL. Future studies should also focus on the long-term assessment of improvements in QoL and explore treatments that target specific subtypes of BMS.

## Appendices

The authors have compiled and summarized the characteristics of the included studies in Table 3.

**Table 3:** Summary of the characteristics of the included studies LLLT, low-level laser therapy; OHIP, Oral Health Impact Profile; SF-36, 36-Item Short Form Survey; IR1W, infrared laser weekly; IR3W, infrared laser three times weekly; OHRQoL, oral health-related quality of life; NAC, N-acetylcysteine; VAS, visual analog scale; GOHAI, Geriatric Oral Health Assessment Index; ppm, parts per million

Reference	Study type	Subjects	Type of treatment	Instrument	Findings
Arduino et al. [24]	Randomized controlled trial	33 patients: 25 females and eight males, divided into two groups. Group A (LLLT): 18 patients, mean age of 68.50±9.31. Group B (clonazepam): 18 patients, mean age of 65.47±7.60	Group A: two laser irradiation sessions weekly for five weeks. Group B: intraoral application of clonazepam (2 mg) for three minutes/three times a day/three weeks	OHIP-49	Group A (LLLT): showed a significant improvement in the assessment of pain, as well as in the quality of life, OHIP-49 score decreased from 59.28±37.95 to 48.22±32.11 after 12 weeks of treatment (p=0.010). Group B (clonazepam): OHIP-49 increased from 63.47±33.69 to 67.87±42.97 after the 12 weeks (p=0.25)
Bardellini et al. [25]	Randomized controlled trial	85 patients: all females, divided into two groups. Group A (LLLT): 43 patients, mean age of 59.76±9.51. Group B (placebo): 42 patients, mean age of 60.86±10.02	Group A: LLLT once a week for 10 weeks. Group B: placebo	OHIP-14	Group A (LLLT): OHIP-14 score went from 16.09±4.2 to 7.34±3.78 at one-month follow-up (p=0.0002). Group B (placebo group): OHIP-14 went from 15.26±3.75 to 10.43±2.99. (p=0.0002). Patients showed an improvement in quality of life related to oral health
Cano-Carrillo et al. [26]	Randomized controlled trial	60 patients: 48 females and 12 males, divided into two groups. Group I: 30 patients, mean age of 61.7±11.6. Group II: 30 patients, mean age was 64.9±14.1	Group I: 30 patients treated with topical lycopene-enriched olive oil (300 ppm), 1.5 mL, three times a day for 12 weeks. Group II: 30 patients taking placebo	OHIP-14 and SF-36	Group I (treatment): OHIP-14 score went from 21 to 18, P-value of 0.035. Group II (placebo): OHIP-14 score went from 23.5 to 18, P-value of 0.035. Significant improvement in oral quality of life assessed through OHIP-14 was found for the two groups, and no significant differences were found between the groups. Regarding the evolution of quality of life, assessed using the SF-36 before treatment and after 12 weeks, intragroup changes were noted across the different domains, although these were not statistically significant except for the domain score relating to corporal pain in the treatment group
de Pedro et al. [27]	Randomized controlled trial	20 patients: 16 females/four males, divided into two groups. Study group: 10 patients, mean age of 60.30±15.19. Placebo group: 10 patients, mean age of 67.60±10.68	Study group: LLLT with a dose of 12 J/cm^2^ during 10 sessions. Placebo group: laser turned off	OHIP-14 and SF-36	Study group: OHIP-14 scores decreased from 16.5±13.09 to 12.5±7.10 at the four-month follow-up. Placebo group: OHIP score increased from 21.30±9.07 to 24.10±11.50 after four months. No significant differences were found in the SF-36 questionnaire for the parameters analyzed
Jurisic Kvesic et al. [28]	Randomized controlled trial	42 patients: four males/38 females divided into two groups. Acupuncture group: 20 patients, two males and 19 females with a mean age of 66.7±12 years. Clonazepam group: 22 patients, two males and 19 females with a mean age of 63.2±14 years	Acupuncture group: received acupuncture for four weeks, three times per week, each session lasting 30 minutes. Clonazepam group: received first clonazepam 0.5 mg once in the morning, for two weeks and after two weeks, twice a day, 0.5 mg in the morning and in the evening, for the next two weeks	SF-36	There were significant changes in the scores of SF-36 (p<0.05). Acupuncture group: increased from 71.6±4.5 to 85.6±5.2. Clonazepam group: increased from 73.4±5.2 to 86±4.7. However, no significant differences were seen between the two therapeutic regimens regarding the SF-36 test
Sikora et al. [29]	Randomized controlled trial	44 participants: one male/43 females, divided into two groups. Mean age of 67.56 years	LLLT group: LLLT was performed with the GaAlAs laser (830 nm), 10 sessions over 10 days. Control group: received placebo	OHIP-14	There were no significant differences between the groups before and after treatment in the quality of life (OHIP-14 scores)
Spanemberg et al. [30]	Randomized controlled trial	78 patients: 11 males/67 females, divided into four groups. Group 1 (IR1W laser): 20 patients with a mean age of 63.6±9.61. Group 2 (IR3W laser): 20 patients with a mean age of 60.5±6.42. Group 3 (red laser): 19 patients with a mean age of 63.2±6.91. Group 4 (control group): 19 patients with a mean age of 61.5±8.76	Group 1 (IR1W laser): LLLT weekly sessions, in a total of 10 sessions. Group 2 (IR3W laser): three LLLT weekly sessions, in a total of nine sessions. Group 3 (red laser): red laser group, three LLLT weekly sessions, in a total of nine sessions. Group 4: control group	OHIP-14	Group 1 (IR1W laser): OHIP-14 score went from 13.77±7.46 to 8.54±5.10. Group 2 (IR3W laser): OHIP-14 score went from 12.87±7.78 to 6.89±4.05. Group 3 (red laser): OHIP-14 score went from 14.46±7.21 to 9.77±4.92. Control group: OHIP-14 score went from 17.80±5.37 to 13.39±3.62 All groups have shown a significant decrease in the OHIP-14 scores. However, only group 2, in which laser was applied three times per week, differed significantly from the control group regarding OHRQoL
Valenzuela and Lopez-Jornet [31]	Randomized controlled trial	44 patients: three males/41 females, divided in three groups. Their average age was 65.5 ranging from 33 to 88 years	Group I (n=16): GaAlAs laser, 815 nm wavelength, 1 W output power, continuous emissions, four seconds; 4 J, and fluence rate of 133.3 J/cm^2^. Group II (n=16): GaAlAs infrared laser, 815 nm wavelength, 1 W output power, continuous emissions, six seconds; 6 J, and fluence rate of 200 J/cm^2^. Group III (n=12): placebo group	OHIP-14	The OHIP-14 values of the subjects in groups I and II presented a relevant improvement during the first 14 days. No significant variation was found when comparing the two laser protocols that were evaluated in this study. Also, no significant changes were observed between days 15 and 28. These findings contrast with group III results, which did not show meaningful improvements in any of the parameters at either time point
Valenzuela, et al. [32]	Randomized controlled trial	57 patients: seven males/50 females divided in two groups. Group A (treatment): mean of 65.8±10.6. Group B (placebo): mean of 67.2±12.6	Group A: 2% chamomile gel. Group B: placebo. Prescription: two times a day/30 days	OHIP-14	There were no significant differences in the OHIP-14 score between the two groups during the comparison period. However, both the treatment and placebo groups exhibited a relevant effect when comparing scores at day 0, two weeks, and one month
Han et al. [33]	Non-randomized controlled trial	160 patients: 18 males/142 females divided into three groups. Group 1: 60 patients, seven males and 56 females with a mean age of 67.0±11.3. Group 2: 37 patients, seven males and 30 females with a mean age of 63.9±15.1. Group 3: 60 patients, four males and 56 females with a mean age of 63.7±11.7	Group 1: NAC 400 mg/day for two months. Group 2: clonazepam 0.5 mg/day for two months. Group 3: NAC+clonazepam for two months	OHIP-14	Group 1: OHIP score decreased from 20.5±13.3 to 18.3±13.7. Group 2: OHIP score decreased from 20.4±12.8 to 16.0±11.4. Group 3: OHIP-14 went from 57.2±25.2 to 36.2±26.9. OHIP-14 scores significantly decreased in all groups. The combination therapy was more effective than either monotherapy
Sardella et al. [34]	Non-randomized controlled trial	10 patients: nine females/one male, mean age of 65.2 years	Acupuncture treatment lasted eight weeks and consisted of 20 sessions	SF-36	No significant improvement in the overall score for the quality of life was observed, although subjects receiving acupuncture treatment seemed better able to cope with their oral symptoms
Kato et al. [35]	Case series	56 patients: their average age was 60.8 ranging from 20 to 83 years	Milnacipran (12 weeks): 30 mg/day and the dosage was raised up to 60 mg and 90 mg/day every four weeks until an improvement of at least 50% reduction in VAS score	SF-36	No significant differences were found in most of the SF-36 items
Sugimoto [36]	Case series	12 patients: all females, mean age of 61.7 years ranging from 47 to 84 years	Milnacipran: 15 mg/day, escalated to 60 mg/day after 28 days, for a total of 84 days (12 weeks)	SF-36 and GOHAI	SF-36 and GOHAI did not vary significantly after treatment
